# Complementary and alternative medicine use among patients with cancer in Mongolia: a National hospital survey

**DOI:** 10.1186/s12906-017-1576-8

**Published:** 2017-01-19

**Authors:** Buyadaa Oyunchimeg, Jung Hye Hwang, Mansoor Ahmed, Soojeung Choi, Dongwoon Han

**Affiliations:** 1 Department of Global Health and Development, Graduate school, Hanyang University, Seoul, South Korea; 2 Institute of Health Services Management, Hanyang University College of Medicine, Seoul, South Korea; 3 Department of Obstetrics and Gynecology, Hanyang University College of Medicine, Seoul, South Korea; 4 Department of Preventive Medicine, Hanyang University College of Medicine, Seoul, South Korea; 5 Department of Preventive Medicine, Hanyang University College of Medicine, 222 Wangsimni-ro, Seongdong-gu, Seoul, 133-791 South Korea

**Keywords:** Cancer, Complementary and alternative medicine, Conventional treatment, Predictors of CAM use, Mongolia

## Abstract

**Background:**

Complementary and alternative medicine (CAM) use is popular in former Soviet Central Asian countries including Mongolia. However, no studies are available on CAM use among patients with cancer in countries of this region. The aim of this research is to describe the prevalence and patterns of CAM use by patients with cancer in Mongolia.

**Methods:**

A cross-sectional study was conducted using data from 482 cancer patients attending the National Cancer Center in Mongolia from September 2015 to February 2016. The survey instrument included 25 questions regarding CAM used, factors associated with use of CAM, cancer-related characteristics, and participants’ socio-demographic profile.

**Results:**

Among 482 respondents (response rate, 95.6%), 47.9% reported using one or more CAM modalities. Products of animal origin were the most popular modalities of CAM, followed by herbal products. Half of the users used CAM while receiving conventional treatment of cancer. Among users, only 29% discussed the CAM use with their doctors. Female gender, younger age, higher education, shorter disease duration, and prior use of CAM were significantly associated with CAM use.

**Conclusions:**

CAM appears to be widely accepted by patients with cancer in Mongolia. The findings support the urgent need for further in-depth study into commonly used oral CAM products and their potential effects on health of patients with cancer in Mongolia. High prevalence of CAM use among cancer patients in our study warrants further studies in other countries of Central Asia.

## Background

The term complementary and alternative medicine (CAM) refers to a wide range of products and practices that are used outside of or alongside with conventional medicine. Globally, the use of CAM is particularly high and increasing among patients with cancer [1–9]. The most pressing concerns associated with such treatments are safety issues especially when they are used along with conventional medicine [10, 11]. In addition, the lack of regulatory oversight raises serious concerns [12].

In the countries of former Soviet Union including Mongolia, the prevalence of CAM use among general population ranges from 3.5 to 38% and many people use CAM for managing symptoms of chronic diseases [13, 14]. Moreover, immigrants from this region who live as ethnic minorities in the Middle East, East Asia and other countries have a high preference for using CAM approaches in several medical conditions [15, 16]. The countries of this region have also one of the highest incidence rates of cancer in the world; however, no studies have focused on the CAM use among patients with cancer [17]. Hence, there is a need to conduct a focused study to provide information that might be useful in the practice, not only to local health professionals but also to the clinicians in those countries where Central Asians particularly Mongolians live as ethnic minorities.

The purpose of this study was to determine the prevalence and patterns of CAM use, especially the specific CAM products used by cancer patients in Mongolia. The study also explored perceptions towards CAM use and predictors for using CAM and its use along with conventional treatment.

## Methods

### Setting and participants

Following the descriptive cross-sectional design, the study was conducted at oncology outpatient clinics (radiotherapy and/or chemotherapy) at National Cancer Center (NCC), the only specialized cancer center in Ulaanbaatar, Mongolia. The National Cancer Center in Ulaanbaatar has both inpatient and outpatient chemotherapy and radiotherapy units where approximately 1300 patients receive the treatment over a period of 6 months. However, for this study only patients attending the outpatient clinics of chemotherapy and radiotherapy were surveyed. Moreover, with the help of hospital staff it was ensured that no patient was surveyed for more than one time in case they visited the clinics again for follow-ups during the study period.

### Survey instrument

After a literature review, the survey instrument appropriate to local context was initially drafted in English and assessed by two experienced oncologists, a pharmacist and a researcher. The questionnaire was then modified according to the assessment, translated into the Mongolian language by a professional translator, and then it was pilot-tested. Translation accuracy of the survey questionnaire was validated by back-translation into English. After this, another pilot test was conducted with 20 cancer patients at different stages of treatment in order to refine the questionnaire and improve its clarity. The final questionnaire comprised four sections. The first section had five questions concerning the individual’s health status, diagnosis, and whether they were currently receiving conventional cancer treatment. The second section consisted of 11 questions related to use of CAM. Questions on patterns of CAM, reasons for use, satisfaction with using CAM and source of information on CAM were only asked from respondents who reported using at least one modality of CAM. The third section contained four questions asking respondent’s attitudes and beliefs toward CAM. The last section dealt with five questions showing the participant’s age, gender, level of income, level of education and area of residence. Conventional cancer treatment, particularly chemotherapy and radiation, are known to cause a number of side effects [18]. After some literature review, a list of five common symptoms, which are routinely experienced by patients with cancer, was included in the survey instrument. All the respondents (CAM users and non-users) were asked to report the symptoms for which they would like to use CAM without a doctor’s prescription.

### Study subjects

The cancer patients attending the oncology outpatient clinic of the hospital from September 1, 2015 to February 28, 2016 were recruited into this study. The inclusion criteria were adult patients (over 20 years old) of either gender with a diagnosis of cancer, aware of their diagnosis of cancer, able to understand the questions, and willing to participate in the study. The ethical approval for this survey was obtained from the School of Medicine at Hanyang University, South Korea (HYI-15-111-2) and the Ministry of Health and Sports, Mongolia (No-05). Enrollment was voluntary and all patients gave written consent to participate in the study.

### Data collection

All patients who met the inclusion criteria during the study period were invited to participate. Information about the research was given verbally to each respondent and consent was sought before the participation. Participants were informed that they could quit the survey anytime they wanted to. For interviewing cancer patients, three experienced interviewers were trained, who explained the CAM to the study participants administered the questionnaire. The patients were given the option either to fill out the questionnaire themselves or to verbally respond to the questions asked by the interviewers. In total, 504 patients agreed to participate in the survey. Of these, 22 were excluded due to an incomplete questionnaire; so the data of 482 participants were entered into the database for further analysis. A high response rate of 95.6% shows the willingness of survey respondents to be helpful as they were ensured complete anonymity.

### Statistical analysis

Respondents were categorized as CAM users or nonusers according to whether they used at least one modality of CAM for cancer-related outcomes since diagnosis. The collected data included socio-demographic and clinical characteristics (type of cancer, present medical treatment, and duration since diagnosis) and CAM use. Comparisons between categorical variables were executed using Pearson’s Chi-square test or Fisher’s exact test. Multivariate logistic regressions were employed for independent variables to evaluate significant factors that could predict use of CAM. *P*-values of < 0.05 were considered statistically significant. All statistical analyses were done using Statistical Package for Social Sciences (SPSS) version 21.0 for Windows.

## Results

### Socio-demographic and clinical characteristics

Sociodemographic and clinical characteristics of the survey participants are presented in Table 1. Out of 482 respondents, the majority were females (63.9%), below 55 years of age (59.5%), from a rural area (66.4%), had less than or equal to high school education (64.1%) and had relatively low monthly household income (≤ $300US/month) (57.1%). The most frequent types of cancer included gastrointestinal (36.1%), urogenital (29%) and breast cancers (15.6%). The majority of patients (*n* = 324; 67.2%) were receiving chemotherapy at the time of study.

**Table 1:** Respondent characteristics of CAM users and non-CAM users

Variables	Total n (%) 482 (100.0)	Non-CAM user n (%) 251 (52.1)	CAM user n (%) 231 (47.9)	*P*-value
Gender				0.001
Male	174 (36.1)	108 (43)	66 (28.6)	
Female	308 (63.9)	143 (57)	165 (71.4)	
Age				0.007
< 55 years	287 (59.5)	135 (53.8)	152 (65.8)	
> 55 years	195 (40.5)	116 (46.2)	79 (34.2)	
Mean age (SD)	51.8 {12.5}	53.1 {12.5}	50.2 {12.4}	<0.012*
Residing area				0.792
Urban	162 (33.6)	83 (33.1)	79 (34.2)	
Rural	320 (66.4)	168 (66.9)	152 (65.8)	
Education level				0.012
No education	4 (0.8)	3 (1.2)	1 (0.4)	
Primary education	29 (6)	19 (7.6)	10 (4.3)	
Middle/high school	281 (58.3)	157 (62.5)	124 (53.7)	
College/university	168 (34.9)	72 (28.7)	96 (41.6)	
Household income				0.105
< 300	275 (57.1)	152 (60.6)	123 (53.2)	
> 300	207 (42.9)	99 (39.4)	108 (46.8)	
Self-perceived health status				0.757
Neither good nor poor	281 (58.3)	148 (59)	133 (57.6)	
Good	201 (41.7)	103 (41)	98 (42.4)	
Type of cancer				0.02
Breast	75 (15.6)	32 (12.)	43 (18.6)	
Gastrointestinal	174 (36.1)	105 (41.8)	69 (29.9)	
Urogenital	140 (29)	73 (29.1)	67 (29)	
Others	93 (19.3)	41 (16.4)	52 (22.5)	
Time since diagnosis				<0.001
0–6 months	385 (66)	221 (88)	167 (71)	
More than 6 months	97 (34)	30 (12)	64 (29)	
Conventional treatment				0.0019
Radiotherapy	96 (19.9)	62 (24.7)	34 (14.7)	
Chemotherapy	324 (67.2)	161 (64.1)	163 (70.6)	
Radiochemotherapy	62 (12.9)	28 (11.2)	34 (14.7)	
Prior use of CAM				<0.001
Yes	232 (48.1)	91 (36.2)	141 (61)	
No	250 (51.9)	160 (63.7)	90 (39)	

### Use of CAM and difference between users and non-users

Use of CAM to manage symptoms associated with cancer and its treatment was reported by 47.9% (*n* = 231) of respondents. Half of users used CAM while receiving conventional cancer treatment. Survey participants more likely to use CAM were female, younger, and had middle/high school education, were more recently diagnosed of cancer, had gastro-intestinal or urogenital cancer, were receiving active chemotherapy, and had used CAM before diagnosis of cancer (Table 1).

### Predictors of CAM use

A logistic regression model was used to assess if any socio-demographic and clinical characteristics were independently associated with the decision to use CAM for cancer-related outcomes (Table 2). The multiple logistic regression model shows that CAM use was significantly associated with female gender (OR 1.749, 95% CI 1.15–2.658, *p* = 0.009), younger age (OR 1.662, 95% CI 1.109–2.493, *p* = 0.014), higher level of education (OR 1.798, CI 1.196–2.702, *p* = 0.005), shorter time since diagnosis (OR 2.963, 95% CI 1.364–6.436, *p* < 0.006), and prior use of CAM (OR 3.027, 95% CI 2.031–4.513, *p* < 0.001).

**Table 2:** Logistic regression model: association of CAM use with various factors

Variable	Category	OR	95% CI		*P* value
Gender	Male	1.749	1.15	2.658	0.009
	Female				
Age	≤55 years	1.662	1.109	2.493	0.014
	>55 years				
Education level	≤ high school	1.798	1.196	2.702	0.005
	Post high school				
Time since diagnosis	≤6 months	2.963	1.364	6.436	0.006
	>6 months				
Prior use of CAM	Yes	3.027	2.031	4.513	<0.001
	No				

### CAM modalities

The frequency of leading CAM modalities along with perceived effectiveness reported by CAM users are listed in Table 3. Products of animal origin (150 of 231 users, 64.9%) were the most popular modalities of CAM used for cancer-related outcomes followed by herbal medicine (32.9%), mind-body therapies (29.4%), dietary supplements (10.8%), and Mongolian traditional medicine (2.2%). Users of products of animal origin reported taking 18 different products while herbal medicine users reported using five different herbs. Of these products, tripe (37.2%), wild animal products (18.2%), rhubarb (18.2%), milk bath (16.9%), and dried foam from a camel’s mouth (16.0%) were most commonly used. Proportion of users who perceived CAM as helpful for the purposes they used for are also presented in Table 3.

**Table 3:** Leading CAM modalities reported by cancer patients^a^

CAM modality	Name of products	N – 231 (%)	N (%) of users who believed the product helped
Products of animal origin (64.9%)	Tripe (goat, sheep etc.)	86 (37.2)	58 (67.4)
	Wild animal products	42 (18.2)	29 (69.0)
	Milk bath	39 (16.9)	31 (79.5)
	Dried foam from camel’s mouth	37 (16.0)	21 (56.8)
	Mare’s milk	1 (0.4)	1 (100.0)
Herbal medicine (32.9%)	Rhubarb	42 (18.2)	18 (42.9)
	Celandine	10 (4.3)	8 (80.0)
	Gardenia	8 (3.5)	4 (50.0)
	Unknown complex herbal capsules or dragees	7 (3.0)	4 (57.1)
	Mushrooms	9 (3.9)	6 (66.7)
	Ginseng	3 (1.3)	2 (66.7)
Mind-body therapies (29.4%)	Mantra	46 (19.9)	36 (78.3)
	Meditation/Yoga	10 (4.3)	8 (80.0)
	Massage	5 (2.2)	4 (80.0)
	Bone setting	3 (1.3)	3 (100.0)
Others	Vitamin/dietary supplements	25 (10.8)	16 (64.0)
	Mongolian traditional medicine	5 (2.2)	4 (80.0)
	Acupuncture	2 (0.9)	1 (50.0)

^a^multiple choice question

### Source of information

As illustrated in Table 4, most CAM users obtained information about CAM modalities from other cancer patients (24.7%), followed by family members (22.5%), friends (19.0%) and mass media and the internet (15.2%). Health professionals including general practitioners and oncologists were least likely to recommend use of CAM (6.4%).

**Table 4:** CAM users’ attitudes and beliefs

Category	N – 231	%
Source of CAM information		
Other patients	57	24.7
Family members	52	22.5
Friends	44	19.0
Mass media/internet	35	15.2
Health professionals	14	6.1
Others	29	12.6
Reasons for using CAM^a^		
To cure the cancer	96	41.6
To boost the immune system	82	35.5
Relief from symptoms	42	18.2
Others	26	11.3
Use of CAM is safe		
Agree	152	65.8
Neutral	54	23.4
Disagree	25	10.8
CAM use and conventional cancer treatment is safe		
Agree	88	38.1
Neutral	57	24.7
Disagree	86	37.2
Disclosure of CAM use to doctor		
Yes	67	29.0
No	164	71.0
Reason for nondisclosure (N – 164)		
Doctor did not ask	124	75.6
Not necessary	31	18.9
Fear that doctor would disapprove	9	5.5

^a^multiple choice question

### Reasons for using CAM

Increasing chance of cure (41.6%) was the most frequently reported reason for using CAM. Patients also associated the consumption of CAM with boosting the immune system (35.5%). Other reasons for using CAM by patients with cancer referred to symptom relief (18.2%) and others (11.3%) (Table 4).

### CAM users’ attitudes and beliefs

Overall, about 65.8% of the CAM users agreed that use of CAM is generally safe, whilst 23.4% were “neutral” and 10.8% disagreed. Moreover, only 38.1% agreed that concurrent use of CAM and conventional treatment of cancer is safe, whereas 37.2% disagreed. Of the 231 users, only 29.0% discussed the concurrent use of CAM with their doctors. Most common reasons for nondisclosure were doctors did not ask (75.6%) and it was not necessary for doctors to know (18.9%) (Table 4).

### CAM use for managing of symptoms

As shown in Table 5, the majority of patients indicated that they would consider CAM use for pain management (38.2%), fatigue (27.0%), constipation (26.3%) and nausea and vomiting (21.6%). Anxiety or depression (17.4%) was least likely to cause use of CAM by patients with cancer.

**Table 5:** Consideration of CAM use for symptom management^a^

Category	Total *n* = 482	CAM user *n* = 231	Non-user *n* = 251
Pain management	184 (38.2)	109 (47.2)	75 (29.9)
Fatigue	130 (27.0)	68 (29.4)	62 (24.7)
Constipation	127 (26.3)	70 (30.3)	57 (22.7)
Nausea/vomiting	104 (21.6)	50 (21.6)	54 (21.5)
Anxiety/depression	84 (17.4)	34 (14.7)	50 (19.9)

N (%)

^a^multiple choice question

## Discussion

To our knowledge, the current findings are the first to report the prevalence and patterns of CAM use among Mongolian patients with cancer. It also described the patients’ attitude and beliefs towards concurrent use of CAM and conventional treatment in oncology setting.

It is believed that Traditional Mongolian Medicine has derived many techniques and theories from the Traditional Tibetan Medicine. It also integrates some aspects of Indian Ayurveda and Traditional Chinese Medicine. In current study, a wide range modalities were included in CAM, and the results show that the prevalence of CAM use (47.9%) is similar to a previous study in Japan [4], but higher than that reported in Europe [6], Australia [8], and South Korea [19]. This difference of CAM utilization rate between several studies could be most likely due to multiple factors such as culture, socioeconomic condition, and/or study methodology itself. Characteristics of CAM users found in this study were in line with previous studies where female gender, younger age, higher level of education, prior use of CAM and shorter time since diagnosis were associated with CAM use [20, 21].

In evaluating types of CAM modalities used by cancer patients, we found that products of animal origin were by far most commonly used. This finding differs from past studies in which vitamins, minerals and herbs were found to be more popular than other types of therapy [6, 7]. Some products of animal origin particularly, raw, fermented or processed dairy products are used as a part of everyday nutrition by many Mongolians. However, based on authors’ understanding of the local culture, some modalities of animal origin were included in CAM because these products are also consumed as medicine. Moreover, in a textbook of Mongolian traditional medicine, Norovsambuu has described clinical indications of several products of animal origin and herbs for a number of diseases including cancer [22]. Additionally, clinical indications of some leading products of animal origin such as tripe have been traced in ancient Chinese dietary medicine texts [23, 24]. According to these texts, tripe has many properties like strengthening the middle burner (spleen and stomach) and restoring qi (life force); thus it is useful for weak people or those who has deficiency of xue (blood) and qi (life force) [25, 26]. However, most of these modalities have gained popularity as CAM based on folk belief and have not yet been evaluated in clinical settings. Consequently, clinicians and in particular oncologists, might encounter unwanted effects and complications among their patients who use such modalities of CAM [10, 11].

We found that nearly half of the CAM users reported “increasing chance of cure”, either directly or indirectly, as the main reason for using CAM. These results are consistent with previous studies [4, 9]; however, other studies that have indicated benefits other than cure (such as, to feel better, to reduce stress and to improve the quality of life) as the main reason for using CAM [27, 28]. The majority of users believed that CAM was effective. However, it is difficult to say whether these improvements were related to use of CAM, since all patients received conventional cancer treatment at the same time. Other patients, family members and friends were a commonly reported source of information about CAM. This finding is in agreement with previous studies investigating the source of information of CAM among cancer patients [6, 29, 30]. Although the questionnaire used in the current study did not ask the patients why they used their social and lay network as a source of information, Verhoef et al. [31] suggested that it may be related to patients’ feeling of connection with these people and thus they consider them as “trusted” source of health information.

Only about 15% of patients in our sample indicated that they would consider CAM use for anxiety and/or depression. This is inconsistent with what is found in previous studies in the US and South Korea, where patients were using multiple CAM modalities to manage depression and anxiety [32–34]. Such an inconsistency could be related to the variety of CAM modalities used by patients with cancer. For instance, there is evidence that patients used yoga therapy to manage anxiety, depression and other emotional symptoms [34–36]. However, yoga therapy was not quite popular among sample of this study.

We also found that most of the CAM users believed CAM to be safe, however, there were split responses when asked about the concurrent use of CAM and conventional treatment. However, all of the CAM users were receiving conventional treatment for cancer. Such results could be due to the social desirability bias, as during the survey the patients were attending a cancer hospital for conventional treatment and few of them might have been afraid to give response in support of CAM. Similar to past studies, a relatively small proportion of CAM users made their doctors aware of their decision to use CAM [3, 5]. This could be dangerous, specifically in the area of oncology, where treatment methods and procedures are becoming more and more advanced. The risk of interaction between patients’ use of CAM by their own decision and conventional treatment might jeopardize their life, as evidence suggests that consumption of CAM can have negative effects when used concurrently with conventional radiotherapy and/or chemotherapy [10, 11]. Therefore, we recommend clinicians to be more aware of their patients’ health care behavior, and more effective communication approaches should be adopted to ensure safe use of CAM especially during conventional cancer treatment.

### Limitations

There are a few limitations of our study. First, as some of the questions investigated past events, so recall bias might have influenced the results. Second, this study might have overestimated the use of CAM among cancer patients as a result of the voluntary response bias. Patients who had an interest in CAM or were using CAM modalities may have been more likely to participate in this study. Moreover, there was a low response from men with cancer and thus the views expressed by the study participants may not fully reflect those of the whole cancer population. As these are the first findings to report use of CAM in Mongolia, a broad range of CAM modalities were included in the survey. The authors are working on another research where Traditional Mongolian Methods of CAM such as bone art and balneotherapy will be reported in near future. Despite these limitations, this study sheds light on different but important aspects of CAM use among Mongolian patients with cancer.

## Conclusions

CAM appears to be widely accepted by patients with cancer in Mongolia. Health care providers should take the initiative to ask whether patients are using any CAM, so they can provide evidence-based consultation regarding the appropriateness of using CAM during conventional cancer treatment. Clinicians in those ethnically and culturally diverse countries where Mongolians are ethnic minorities should be aware of the fact that many Mongolian cancer patients use modalities of CAM for cancer-related outcomes. The findings support the urgent need for further in-depth study into commonly used CAM products and their potential effects on health of cancer population in Mongolia. High prevalence of CAM use among patients with cancer in our study warrants further studies in other countries of this region.

## Abbreviation

CAM: Complementary and alternative medicine
